# Dataset of the rumen microbiota and epithelial transcriptomics and proteomics in goat affected by solid diets

**DOI:** 10.1038/s41597-024-03584-7

**Published:** 2024-07-10

**Authors:** Jianmin Chai, Xiaokang Lv, Yimin Zhuang, Qiyu Diao, Kai Cui, Feilong Deng, Ying Li, Naifeng Zhang

**Affiliations:** 1 Guangdong Provincial Key Laboratory of Animal Molecular Design and Precise Breeding, College of Life Science and Engineering, Foshan University, Foshan, 528225 China; 2 Institute of Feed Research of Chinese Academy of Agricultural Sciences, Key Laboratory of Feed Biotechnology of the Ministry of Agriculture and Rural Affairs, Beijing, 100081 China; 3 Department of Animal Science, Division of Agriculture, University of Arkansas, Fayetteville, AR 72701 USA; 4 Anhui Province Key Laboratory of Animal Nutritional Regulation and Health, College of Animal Science, Anhui Science and Technology University, Chuzhou, China

**Keywords:** Bacterial host response, Proteomics, Transcriptomics

## Abstract

Although early solid diet supplementation is a common practice to improve the growth and development in goat kids, its biological mechanism how solid diet induces rumen microbiota and epithelial development is still unknow. In this study, rumen fermentation parameters, 16S rRNA sequencing for rumen content and epithelial microbiota, transcriptomics and proteomics of epithelium were determined to classify the effects of solid diet supplementation. Here, we classified the changes of goat phenotypes (i.e., growth performance, rumen fermentation and development) and linked them to the changes of rumen microbiota, transcriptome and expressed proteins. The mechanism of solid diet improving rumen development was elucidated preliminarily. Moreover, different roles between the rumen content and epithelial microbiota were identified. Thess datasets expands our understanding of the association between the early diet intervention and rumen development, providing the useful information how nutrient strategy affects rumen function and subsequently improves the host growth. The generated data provides insights in the importance of rumen niche microbiota and microbe-host interactions, which benefits future studies.

## Background & Summary

As one of the most important livestock specie in the world, the goat (*Capra hircus*) provides various types of products for human consumption, such as meat, milk, pelts and fiber^1,2^. Now, there are over 1,000 goat breeds and total goat amount is over 1 billion globally^3^. The rumen, the most critical digestive organ for ruminants, can degrade the high fiber-based plant components into volatile fatty acids (VFA) and microbial protein for rumen development and body nutrient requirement via microbial fermentation process^4^. The ability to digest fiber is the evidence of the well-developed and functional rumen in young ruminants^5^. However, the rumen of goat kids develops rapidly after born. Manipulation the early development of rumen becomes the most effective way to improve life-long rumen function and animal growth^6,7^. Early supplementation of a concentrate diet has already been widely used in ruminant production to improve its rumen and body development because of its stimulation of microbial proliferation and VFA production that initiates epithelial development^8–11^. However, the biological mechanism of this practise is still unclear.

**Fig. 1:**
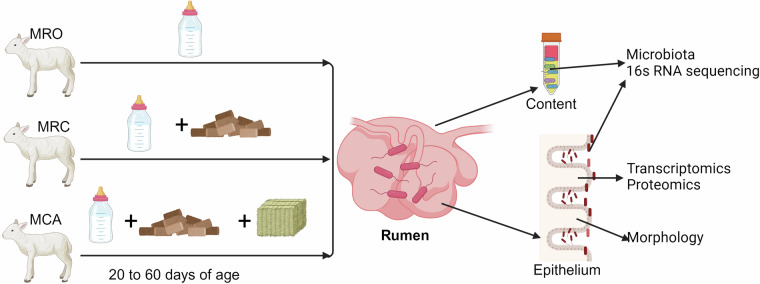
Overview of the experimental workflows. The goat kids were assigned into three treatments (milk replacer only (MRO), milk replacer supplemented concentrate (MRC) and milk replacer supplemented concentrate plus alfalfa pellets (MCA)) on 20 days of age. At the end of animal feeding trial (60 days of age), goat kids were slaughtered for rumen sample collection. After the rumen was weighted, rumen content and epithelial microbial samples were collected for 16S rRNA sequencing. The rumen epitheliums were collected for transcriptomics, proteomics, and morphology measurements.

Previous studies have demonstrated that early feeding starter with high grains or even alfalfa changed the rumen microbiota and improved animal growth^12–14^. However, most studies focused on the rumen content microbiome until now, and less researches have been conducted to determine the microbiota attached on the rumen epithelium and the rumen epithelial transcriptomics and proteomics. The rumen epithelial microbiota associated with the content microbial community might be critical for nutrient absorption as it tightly attaches on the luminal side of the rumen^15–17^. A previous study found differing community structure between the rumen content and the epithelial microbiome in cattle^18^, and the epithelial microbiota was also affected by dietary carbohydrate^17^. Moreover, the import roles of epithelial microbiota in maintaining host gene expression and development were also reported^19^. Beyond microbiota, changes of rumen fermentation and epithelial genes were reported in previous studies^4,17,20,21^. It is known that rumen epithelium plays key role in digestion and absorption of nutrients, such as VFAs and ammonia^22^. Thus, understanding the regulations of rumen epithelial gene and protein expression affected by the early diet intervention is necessary and urgent. Additionally, limit in microbe-host interactions develops a gap for understanding the connection between microbiota and rumen development as well as the goat growth. Therefore, this study was conducted to investigate the 16S rRNA gene sequences of rumen microbiota (both content and epithelium) and host transcriptomics and proteomics in goat kids consuming three diet regimes: milk replacer only (MRO), milk replacer supplemented with concentrate solid diet (MRC), and milk replacer supplemented with concentrate diet and alfalfa (MCA). This dataset, including the goat kids’ phenotypes, rumen content and epithelial microbiota, and epithelial omics (both transcriptome and proteomics), was described to illustrate the effects of early supplementation of high carbohydrates on the goat kids and explored the axis of diet-microbiota-host. As a foundation data, these omics could allow us to dig more relationship between the rumen microbiome and epithelial genes. The details of a schematic overview of the study workflow were shown in Fig. 1.

## Methods

### Ethical statement

All experimental animals’ procedures in this study were approved by the Chinese Academy of Agricultural Sciences Animal Ethics Committee.

### Experimental design, animal management and sampling

Based on the experimental design, 72 healthy Haimen goat kids (4.53 ± 0.52 kg body weight (BW)) were assigned into three treatment group: milk replacer only (MRO), milk replacer supplemented concentrate (MRC) and milk replacer supplemented concentrate and alfalfa pellets (MCA). Six animal replicates were included in each treatment group. Goat kids consumed these diet regimes from 20 to 60 days of age (d), respectively, and were slaughtered on d 60 to collect samples.

The animal trial was conducted at a commercial farm (the Green Sheep Valley Farm, Haimen City, China). During the trial, all goat kids had free access to water. Milk replacer, a patent product, was obtained from Beijing Precision Animal Nutrition Research Center, China. The solid diet, both concentrate and alfalfa pellets, were freely provided the MRC and MCA groups.

The feed intake was recorded daily and is shown in Table 1. At d 60, six goat kids from each treatment were weighted and slaughtered to collect rumen samples. Approximately 10 mL of rumen content was sampled from the mixed digesta and stored at −80 °C for next-generation sequencing. Rumen fluid phase approximately the 10 mL level was filtered via four layers of gauze and stored in a 15 mL tube at −20 °C for the analysis of rumen fermentation parameters. Next, rumen tissue at the bottom of the ventral sac was washed using sterilized PBS (pH = 7) to rinse the residual of rumen content or fluid filling the gap between papillae. Remaining residues attached tightly in the epithelium were abraded out to analyze for the epithelial microbiota. Concurrently, tissue sections (~4 cm^2^) in the ventral sac were fixed in a solution of 10% formalin for epithelial morphology detection. Samples for the remaining tissue and the epithelium-associated microbiota were snap-frozen in liquid nitrogen and stored at −80 C for host transcriptome and proteomics, respectively.

**Table 1:** Effect of early supplementary solid diet on nutrient intake of goat kids.

Items	Treatments			SEM	*P* value
	MRO	MRC	MCA		
Milk replacer intake /g•d^−1^	130.62	107.91	119.9	4.95	0.1696
Concentrate intake /g•d^−1^	—	188.18	189.56	4.82	0.8956
Alfalfa pellets intake /g•d^−1^	—	—	34.34	—	—
Dry matter intake /g•d^−1^	122.47^c^	271.50^b^	309.97^a^	16.12	<0.0001
Protein intake /g•d^−1^	31.33^c^	58.52^b^	66.37^a^	4.07	<0.0001
NDF-Neutral detergent fiber intake /g•d^−1^	—	49.25^b^	66.08^a^	3.13	0.0004
NFC-Non-fibrous carbohydrate intake /g•d^−1^	63.82^c^	118.16^b^	131.15^a^	7.94	<0.0001

MRO = milk replacer, MRC = milk replacer + concentrate, MCA = milk replacer + concentrate + alfalfa; SEM = Standard error of the means.

In the same row, values with different small letter superscripts mean significant difference (P < 0.05). The superscripts alphabet order represents a decreasing order of the mean among groups.

### Rumen fermentation parameters measurement

Determination of the NH_3_-N using a phenol-sodium hypochlorite colorimetric method was performed after the rumen liquid was thawed at 4 °C. Rumen microbial proteins were analyzed according to the method described by Makkar *et al*.^23^. VFA concentration was quantified by gas chromatography (GC)^24^ using methyl valerate as the internal standard in an Agilent 6,890 series GC equipped with a capillary column (HP-FFAP19095F-123, 30 m, 0.53 mm diameter and 1 mm thickness). The detection results of rumen fermentation parameters are shown in Table 2.

**Table 2:** Effect of early feeding on rumen fermentation parameters of goat kids.

Items	Treatments			SEM	*P* value
	MRO	MRC	MCA		
pH	7.09^a^	5.88^b^	6.01^b^	0.15	<0.0001
NH_3_-N, mg/dL	19.61^a^	6.02^b^	5.40^b^	1.84	<0.0001
Microbial protein, mg/mL	0.02^b^	1.07^a^	1.24^a^	0.15	0.0006
Total VFA, mmol/L	34.99^c^	76.53^b^	91.40^a^	6.28	<0.0001
Acetate, mmol/L	22.27^c^	38.75^b^	45.45^a^	2.64	<0.0001
Propionate, mmol/L	9.45^b^	17.54^a^	22.06^a^	1.64	0.0014
Isobutyrate, mmol/L	0.24	0.48	0.23	0.08	0.3589
Butyrate, mmol/L	1.74^c^	11.39^b^	17.73^a^	1.84	<0.0001
Isovalerate, mmol/L	0.56	0.98	0.91	0.16	0.547
Valerate, mmol/L	0.73^b^	7.34^a^	5.01^a^	0.95	0.0045
Acetate:Propionate	2.55	2.23	2.2	0.13	0.5502

VFA = volatile fatty acids

In the same row, values with different small letter superscripts mean significant difference (*P* < 0.05). The superscripts alphabet order represents a decreasing order of the mean among groups.

### Measurement of rumen epithelial morphology

Rumen tissue sections were kept in 70% ethanol until further measurement after 24 h of fixing in formalin. All samples were stained with Yihong-hematoxylin (H.E.) at the Chinese Agriculture University (Beijing, China). The length and width of the rumen papillae and stratum corneum thickness were measured using the Axiovision software (Zeiss, Oberkochen, Germany) Image-pro express image analysis processing system. The results of rumen papilla length, papilla width, lamina propria thickness and epithelial thickness are displayed in Table 3.

**Table 3:** Effects of early supplementary solid diet on growth performance and rumen fermentation parameters in goat kids.

Items	Treatments			SEM	*P* value
	MRO	MRC	MCA		
Slaughter BW/kg	7.01^b^	10.47^a^	10.23^a^	0.41	<0.0001
Average daily gain/g	70.28^b^	123.93^a^	126.25^a^	5.48	<0.0001
Rumen weight/g	47.42^c^	126.70^b^	175.67^a^	14.94	<0.001
Papilla length/μm	786.6^b^	1191.6^a^	1389.6^a^	80.44	0.0008
Papilla width/μm	169.81^b^	206.52^ab^	237.13^a^	9.85	0.0096
Keratin layer thickness/μm	33.68	49.98	36.75	3.38	0.1258
Muscle layers thickness/μm	698.6	628.4	861.3	47.28	0.1171
Epithelium thickness/μm	43.13^b^	56.07^a^	47.72^ab^	2.19	0.04

MRO = milk replacer, MRC = milk replacer + concentrate, MCA = milk replacer + concentrate + alfalfa; SEM = Standard error of the means. BW = body weight; A:P = the ratio of acetate and propionate

In the same row, values with different small letter superscripts mean significant difference (*P* < 0.05). The superscripts alphabet order represents a decreasing order of the mean among groups.

### Next-generation sequencing of rumen content and epithelial microbiota and analysis

Total rumen content and epithelial microbial DNA were extracted using the Magnetic Universal Genomic DNA Kit (QIAGEN Inc., Beijing, China) according to the manufacturer’s protocol, and the V3-V4 region of the bacterial 16S ribosomal RNA genes was amplified using adaptor-linked universal primers (341 F and 806 R). The concentration of DNA was determined using Qubit^®^ DNA Assay Kit with a Qubit^®^ 3.0 Fluorometer (Invitrogen, China). Amplicon libraries were built using all qualified products and sequenced with an Illumina HiSeq PE250 platform at the Realbio Technology Genomics Institute (Shanghai, China). More details related to the sequencing process can be found in our previous study^21^.

Raw sequencing files of the rumen content and epithelial microbiota were processed using the mothur program (v1.39.1)^25^. Forward and reverse reads were merged first, and low-quality reads were removed. The high-quality sequences were then aligned against the SILVA reference database (Full-length sequences and taxonomy references release 132, http://www.arb-silva.de/)^26^. Moreover, the VSEARCH algorithm was employed to remove chimeras in filtered sequences. Subsequently, high-quality sequences were clustered into operational taxonomic units (OTUs) at the 97% similarity level using the Ribosomal Database Project (RDP) database^27^. Alpha (Shannon Index and Observed OTUs) and beta diversities (Bray-Curtis and Jaccard distance) were calculated using mothur. The boxplots of alpha diversity and the PCoA plot of beta diversity were visualized using the ‘ggplot2’ package in R (v3.6.0). The ANalysis Of SIMilarity (ANOSIM) test was performed to test the statistical significance of beta diversity.

### Transcriptomic profile of the rumen epithelial tissue

Total RNA of the rumen epithelial samples was extracted using the TRIzol reagent (Invitrogen, CA, USA). The RNA integrity was measured using an Agilent 2100 Bioanalyzer (Agilent, Santa Clara, CA, USA). If samples had the RNA integrity equal to or over 7, it would be used for sequencing. Then, the library was prepared and sequenced at the Beijing Genomics Institution (Shenzhen, China) using the HiSeq2000 system (Illumina, CA, USA) to obtain 100-bp paired-end reads according to the manufacturer’s instructions.

Raw reads were filtered to obtain clean reads using the trimmomatic module in SOAPnuke (v1.4.0) software via the removal of adaptors and low-quality reads. Low-quality reads were defined as more than 20% of bases with a quality score smaller than 10 or having more than 5% ambiguous sequences labeled as “N”. Then, high-quality RNA reads were mapped and assembled to reference genomes (AnimalTFDB v2.0) using HISAT (v2.1.0)^28^. The detection of transcript expression levels was based on the number of fragments per kilobase of exon per million fragments mapped (FPKM). Differentially expressed genes (DEGs) were detected based on methods reported by Wang *et al*.^29^ and the false discovery rate (FDR) was calculated based on methods of Benjamini and Hochberg’s multiple testing correction^30^. The significantly DEG were confirmed at a fold change ≥ 2 and a false discovery rate (FDR) < 0.001. Using this method, the DEG were displayed through a pairwise comparison analysis (MRO-vs-MRC, MRO-vs-MCA and MRC-vs-MCA). After expression pattern clustering, the DEG from pairwise comparisons were subjected to functional annotation, including GO (Gene Ontology) functional annotation and KEGG (Kyoto Encyclopedia of Genes and Genomes) pathway annotation. The GO terms and KEGG pathway enrichment were performed using The Database for Annotation, Visualization and Integrated Discovery (DAVID v 6.8, http://david.abcc.ncifcrf.gov)^31^.

### Proteomics of the rumen epithelium

Proteins from epithelial samples were extracted using Lysis buffer 3 (8 M Urea, 40 mM Tris-HCl or TEAB with 1 mM PMSF, 2 mM EDTA and 10 mM DTT, pH 8.5) and two magnetic beads (diameter 5 mm). Then, mixtures were placed into a TissueLyser to release proteins. After centrifugating, the supernatant was transferred into a new tube, reduced with 10 mM dithiothreitol (DTT) at 56 °C for 1 hour and alkylated by 55 mM iodoacetamide (IAM) in the dark place at room temperature for 45 min. After a new round of centrifugation (25,000 g, 4 °C, 20 min), the supernatant was quantified by Bradford. Moving to next step of quality control of protein extraction, we then mixed 15–30 μg proteins with loading buffer in centrifuge tube and heated them at 95 °C for 5 minutes (min). Then, the supernatant was centrifuged at 25,000 g for 5 min and loaded to sample holes in 12% polyacrylamide gel. The SDS-PAGE in constant voltage was performed to detect proteins quality at 120 V for 120 min. Once finished, it was stained in gel with Coomassie Blue for 2 hours and added destaining solution (40% ethanol and 10% acetic acid). Ultimately, it was put on a shaker (exchange destaining solution for 3~5 times, 30 min a time). Next, the protein digestion step needed that the protein solution (100 μg) with 8 M urea was diluted 4 times with 100 mM TEAB. Then the proteins were digested at 37 °C overnight by Trypsin Gold (Promega, Madison, WI, USA) in a ratio of protein: trypsin = 40:1. After trypsin digestion, the peptides were desalted using Strata X C18 column (Phenomenex) and vacuum-dried according to the manufacturer’s protocol. Then, we did protein labeling. The peptides were dissolved in 30 μL 0.5 M TEAB with vertexing. After the iTRAQ labeling reagents were recovered to ambient temperature, they were transferred and combined with proper samples. Peptide labeling was performed by iTRAQ Reagent 8-plex Kit according to the manufacturer’s protocol. The labeled peptides with different reagents were combined, desalted with a Strata X C18 column (Phenomenex), and vacuum-dried according to the manufacturer’s protocol. Subsequently, peptide fractionation step was carried out. A Shimadzu LC-20AB HPLC Pump system coupled with a high pH RP column was employed for the separation of peptides. The peptides were reconstituted with buffer A (5% ACN, 95% H_2_O, adjust pH to 9.8 with ammonia) to 2 ml and loaded onto a column containing 5 μm particles (Phenomenex). The peptides were separated at a flow rate of 1 mL/min with a gradient of 5% buffer B (5% H_2_O, 95% ACN, adjusted pH to 9.8 with ammonia) for 10 min, 5–35% buffer B for 40 min, 35–95% buffer B for 1 min. The system was then maintained in 95% buffer B for 3 min and decreased to 5% within 1 min before equilibrating with 5% buffer B for 10 min. Elution was monitored by measuring absorbance at 214 nm, and fractions were collected per 1 min. The eluted peptides were pooled into 20 fractions and vacuum dried. Next, each fraction was resuspended in buffer A (2% ACN, 0.1% FA) and centrifuged at 20,000 g for 10 min. The supernatant was loaded onto a Thermo Scientific™ UltiMate™ 3000 UHPLC system equipped with a trap and an analytical column. The samples were loaded on a trap column at 5 μL/min for 8 min, and then eluted into the homemade nanocapillary C18 column (ID 75 μm × 25 cm, 3 μm particles) at a flow rate of 300 nl/min. The gradient of buffer B (98% ACN, 0.1% FA) was increased from 5% to 25% in 40 min, and then increased to 35% in 5 min, followed by 2 min linear gradient to 80%, then maintained at 80% B for 2 min, and finally returned to 5% in 1 min and equilibrated for 6 min. Finally, we used Mass Spectrometer to detect the proteins. The peptides separated from nanoHPLC were subjected into the tandem mass spectrometry Q EXACTIVE HF X (Thermo Fisher Scientific, San Jose, CA) for DDA (data-dependent acquisition) detection by nano-electrospray ionization. The parameters for Mass Spectrometer (MS) analysis were listed as following: electrospray voltage: 2.0 kV; precursor scan range: 350–1500 m/z at a resolution of 60,000 in Orbitrap; MS/MS fragment scan range: > 100 m/z at a resolution of 15,000 in HCD mode; normalized collision energy setting: 30%; dynamic Exclusion time: 30 s; Automatic gain control (AGC) for full MS target and MS2 target: 3e6 and 1e5, respectively. The MS/MS scan numbers followed one MS scan: 20 most abundant precursor ions above a threshold ion count of 10,000.

The raw MS/MS data was converted into Mascot Generic File (MGF) format, and the MGF files were searched by the local Mascot server against the database. Besides, quality control was performed to determine if a reanalysis step was needed. An automated software, called IQuant, was applied to analyze the labeled peptides with isobaric tags, with steps of protein identification, tag impurity correction, data normalization, missing value imputation, protein ratio calculation, statistical analysis, results presentation. All proteins with a false discovery rate (FDR) less than 1% will proceed with downstream analysis.

## Data Records

The raw reads files for each rumen content sample of 16S rRNA sequencing have been uploaded to the NCBI Sequence Read Archive (SRA) with accession number SRP199804^32^, and the raw data of the rumen epithelial samples of 16S rRNA sequencing and transcriptomics have been deposited into NCBI SRA with accession number SRP236061^33^. The raw proteomics data were uploaded to ProteomeXchange Consortium via the iProX partner repository with the dataset identifier PXD047843^34^. All these data can be used freely.

## Technical Validation

Benefits of early supplementation of high carbohydrate diet was found in this dataset. As shown in Tables 1 and 2, significant increases in nutrient intake, average daily gain and body weight were observed in MRC and MCA groups. Moreover, compared to MRC, MCA had a higher intake of protein, neutral detergent fibres (NDF), and non-fibrous carbohydrates (NFC). Next, a more well-developed rumen was also found in solid diet groups as rumen weight, papilla length and width were significantly increased in MRC and MCA groups. We found that the parameters of rumen fermentation were also affected by solid diet supplementation (Table 3). Compared to MRO, lower NH_3_-N concentration was found in MRC and MCA, while higher concentrations of total VFA, acetate, propionate, butyrate and valerate in MRC and MCA were observed.

**Fig. 2:**
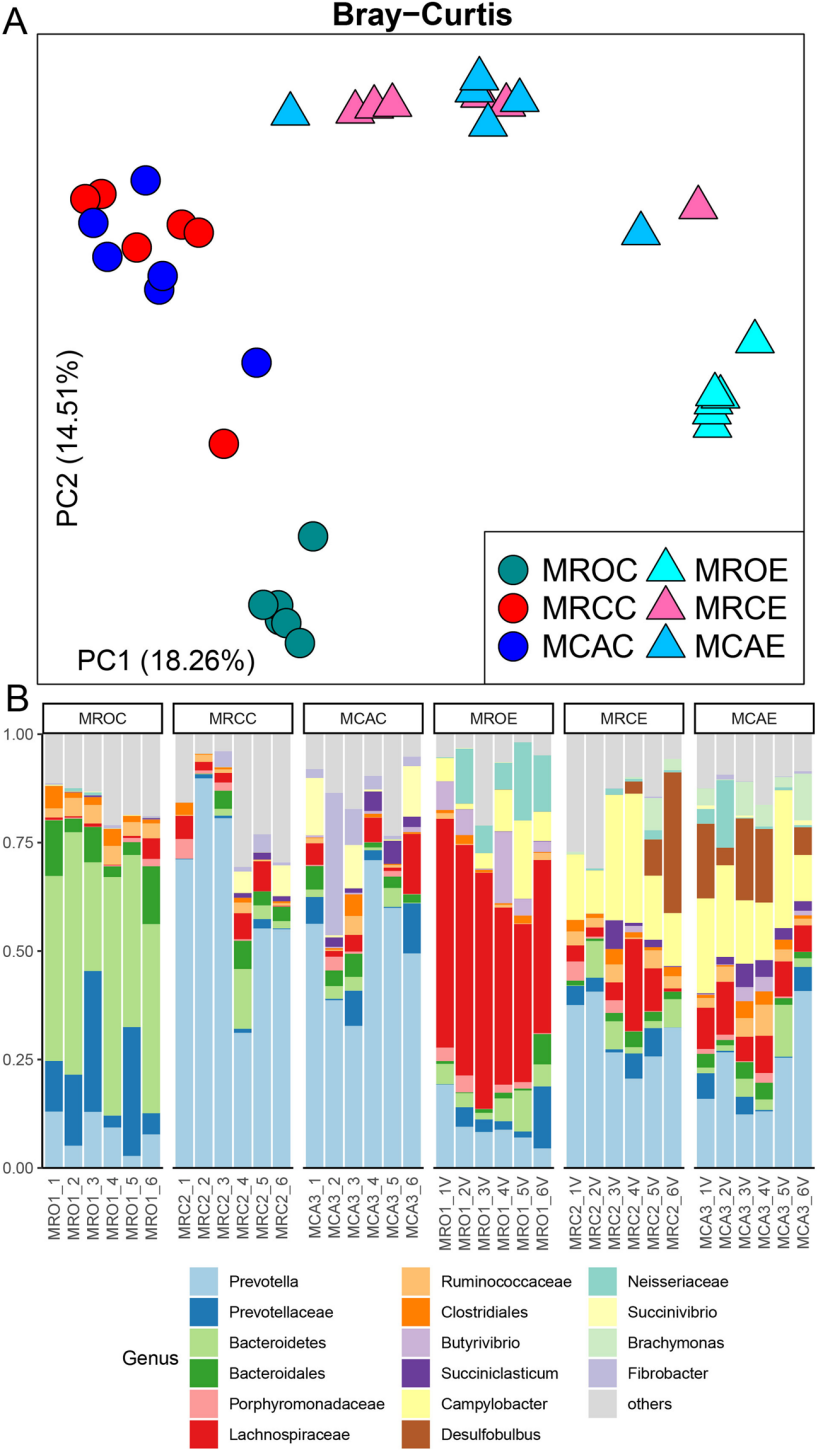
Next-generation sequencing of the rumen content and epithelial microbiota in goat kids. (**A**) Beta diversity of the rumen content and epithelial microbiota based on Bray–Curtis. One point represents one sample. (**B**) Rumen microbial composition at the genus level. Each column represents a sample, and each bar represents one bacterium. MROC, MRCC and MCAC represent content samples in animals that received MRO, MRC and MCA diets, while MROE, MRCE and MCAE represent the epithelial microbiota from the three diets, respectively. The MRO treatment was fed only milk replacer, the MRC treatment was fed milk replacer with concentrate and the MCA treatment was fed milk replacer with concentrate plus alfalfa.

For next-generation sequencing, the DNA quality of the 16S was determined, and the DNA total amount ≥ 1 μg and concentration ≥ 30 ng/μL indicated that the DNA quality was qualified. The concentration of metagenome libraries was assessed using an Agilent 2100 Bioanalyzer instrument (Agilent DNA 1000 Reagents) and a Genomic DNA Sample Prep Kit for Illumina NovaSeq 6000 Platform, and the libraries with qualified concentration (≥10 nM) and volume (15 μL–100 μL) were subjected for sequencing. Quality control of 16S rRNA sequencing reads was performed using mothur MiSeq SOP (https://mothur.org/wiki/miseq_sop/). The quality assessment of 16S rRNA sequencing reads of both rumen content and epithelial samples is shown in Supplementary Table 1. As shown in Fig. 2A, the samples of rumen content and epithelium tended to cluster based on the organism (mainly along the first axis), the second factor of variation being the individual intra-species variability (y-axis). Thus, PCoA separated the samples according to their origin. The bacteria, including *Prevotella* and *Bacteroidetes*, dominated the rumen content communities, while epithelial samples had higher abundances of *Prevotella*, *Lachnospiraceae unclassified*, *Campylobacter*, and *Desulfobulbus* (Fig. 2B).

**Fig. 3:**
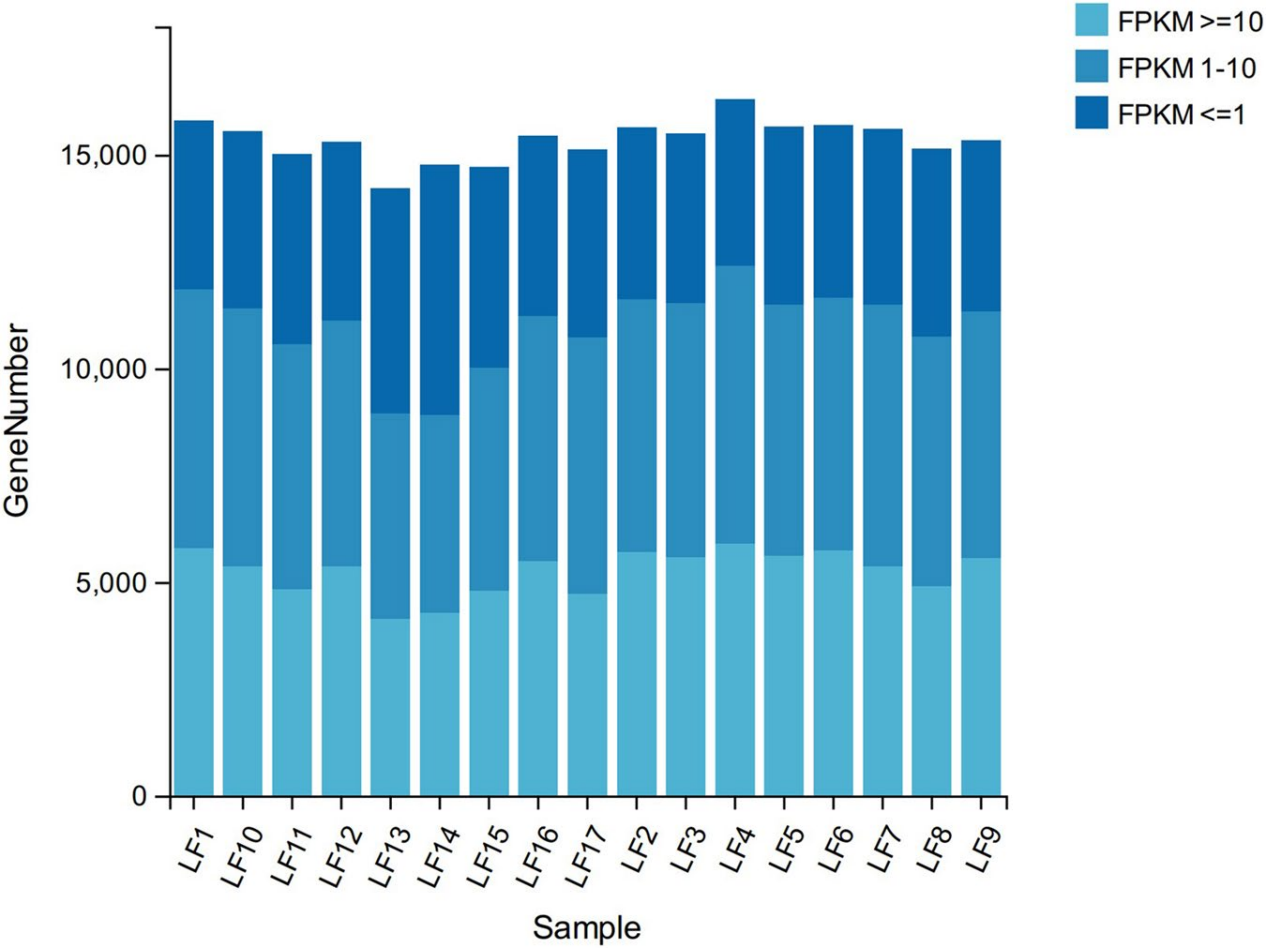
Gene expression stacked bar plot of each epithelial sample. LF1-LF6, LF7-LF12 and LF13-LF17 belong to MRO, MRC and MCA treatments, respectively. MRO = milk replacer, MRC = milk replacer + concentrate, MCA = milk replacer + concentrate + alfalfa.

To ensure the quality of the transcriptomic sequencing data, a state-of-the-art equipment for molecular biology was employed to determine the purity, concentration, and integrity of RNA. Subsequently, the library’s quality was assessed through testing. Once the requirements are met, computer sequencing can be conducted. A total of 109 Gb of clean data were generated, with an average of 6.44 Gb per subject. After filtration using trimmomatic, the proportion of clean reads with quality score over 30 was 96.79% (Supplementary Table 2). When mapping the high-quality reads to reference genome, we found the average of the mapping rations of all samples was 76.75% (Supplementary Table 2). The gene expressions of each epithelial sample were shown in Fig. 3.

**Fig. 4:**
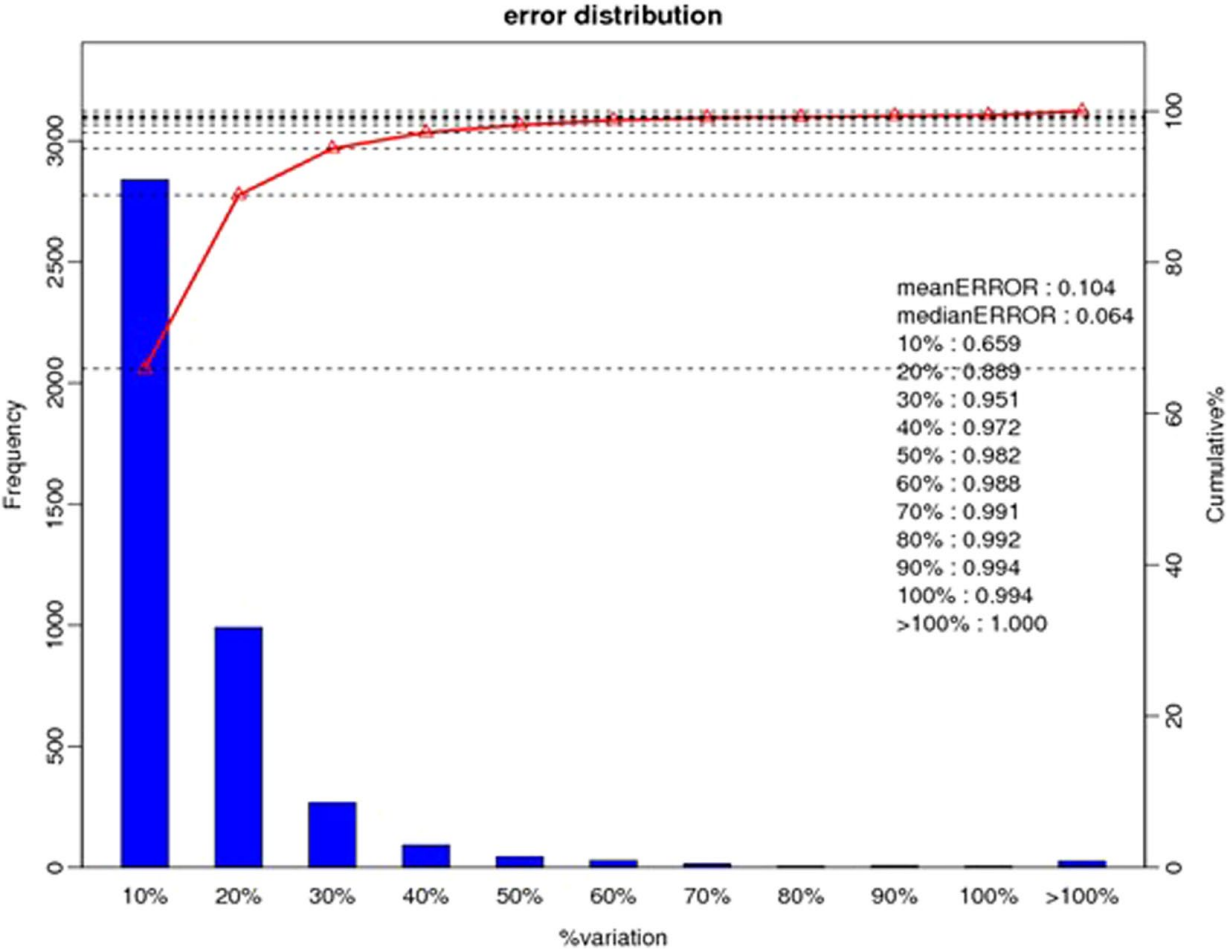
Quantification repeat analysis of the rumen epithelial proteomics. X-axis is the deviation between the protein ratio of the repetitive samples. Y-axis is the quantified protein amount at the corresponding range.

**Fig. 5:**
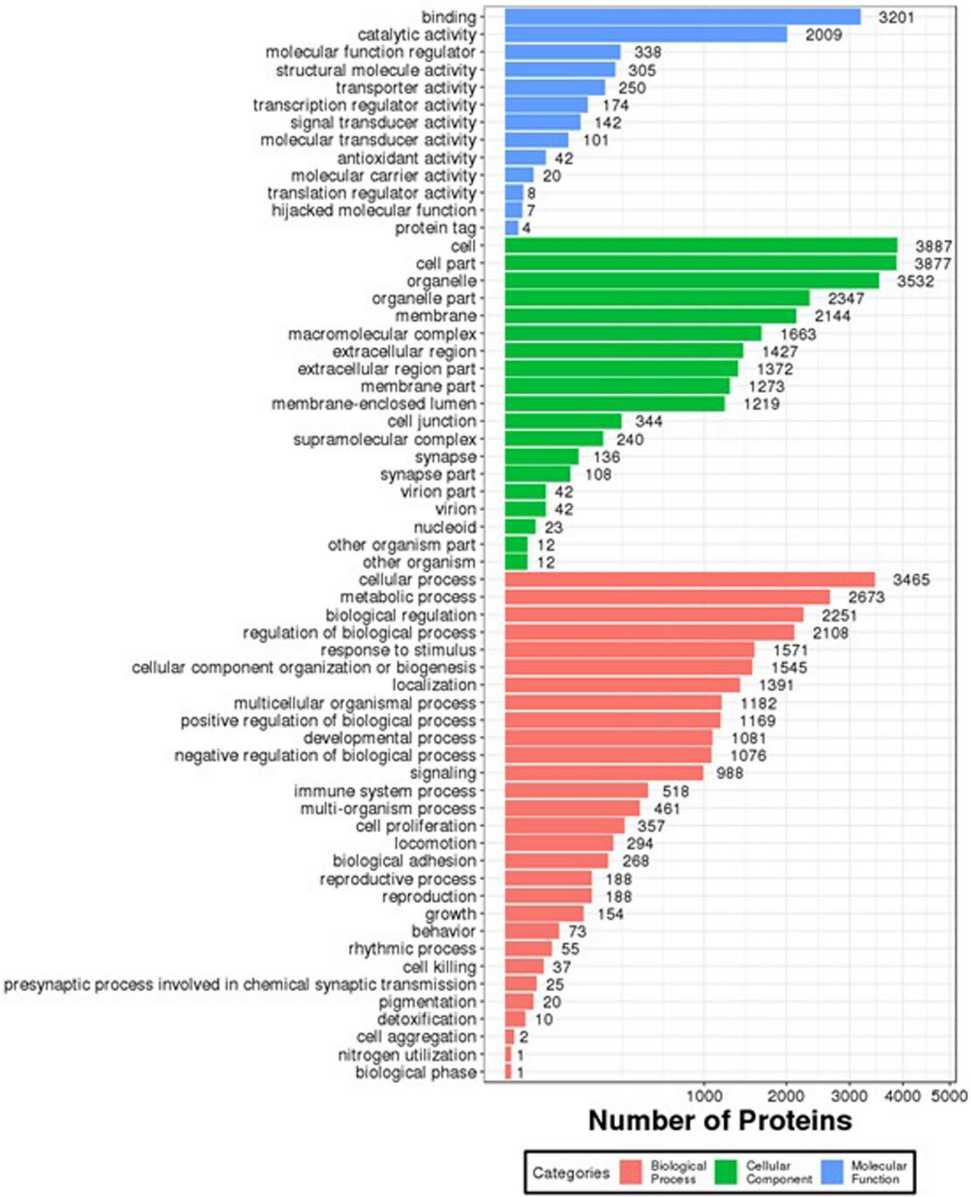
Bar plot of the Gene Ontology Analysis using proteomics. The bar chart shows the distribution of corresponding GO terms. Different colors represent different GO categories.

The iTRAQ (Isobaric tags for relative and absolute quantitation, iTRAQ) technology was confirmed to have its high precision in protein quantitative method. Three technical duplicate experiments were conducted for each sample. Totally 1,443,120 spectrums were generated, 26,793 peptides and 6,003 proteins were identified with 1% FDR. Coefficient of Variation (CV) defined as the ratio of the standard deviation (SD) to the mean was used to evaluate the reproducibility (Fig. 4). The lower the CV, the better the reproducibility. The Gene Ontology (GO) annotation for all identified proteins were displayed (Fig. 5).

## Usage Notes

Our comprehensive dataset of rumen microbiota and epithelial omics resulting from solid diet provides insights into association between the critical microbiota and host gene expression. Although our preliminary findings reveal how solid diet and its nutrients drive rumen microbiome, epithelial gene and proteins, more interesting biological pathways can be dug via re-analysing our omics dataset, which allow us to deeply understand the interactions between microbiome and host. Moreover, common bioinformatic software and pipeline used in this study is great for reuse of the data.

### Supplementary information

supplemental table s1

supplemental table s2

## Data Availability

Serum enzyme activity, antioxidant capacity, and immune parameter were statistically processed and analyzed using R version 3.6.0, default parameters or parameters recommended by the developer were used. A reproducible version of partial microbiome analysis and computer code is available at https://github.com/chaichai9521/goat-rumen-mcirobiome-analysis.git.

## References

[1] Cao Y (2023). The multi-kingdom microbiome of the goat gastrointestinal tract. Microbiome.

[2] Mizrahi I, Wallace RJ, Morais S (2021). The rumen microbiome: balancing food security and environmental impacts. Nat Rev Microbiol.

[3] Dong Y (2013). Sequencing and automated whole-genome optical mapping of the genome of a domestic goat (Capra hircus). Nat Biotechnol.

[4] Chai J (2021). Solid diet manipulates rumen epithelial microbiota and its interactions with host transcriptomic in young ruminants. Environ Microbiol.

[5] Chai J, Zhuang Y, Cui K, Bi Y, Zhang N (2024). Metagenomics reveals the temporal dynamics of the rumen resistome and microbiome in goat kids. Microbiome.

[6] Eisler MC (2014). Agriculture: Steps to sustainable livestock. Nature.

[7] Chai J (2024). Diet and monensin influence the temporal dynamics of the rumen microbiome in stocker and finishing cattle. J Anim Sci Biotechnol.

[8] Sun DM, Mao SY, Zhu WY, Liu JH (2018). Effect of starter diet supplementation on rumen epithelial morphology and expression of genes involved in cell proliferation and metabolism in pre-weaned lambs. Animal.

[9] Vi RLB, McLeod KR, Klotz JL, Heitmann RN (2004). Rumen development, intestinal growth and hepatic metabolism in the pre-and postweaning ruminant. J Dairy Sci.

[10] Heinrichs, A. J. Rumen development in the dairy calf. *Calf and heifer rearing*, 53-65 (2005).

[11] Suarez BJ (2006). Effects of supplementing concentrates differing in carbohydrate composition in veal calf diets: I. Animal performance and rumen fermentation characteristics. J Dairy Sci.

[12] Yang B, He B, Wang SS, Liu JX, Wang JK (2015). Early supplementation of starter pellets with alfalfa improves the performance of pre- and postweaning Hu lambs. J Anim Sci.

[13] Yang B (2018). Alfalfa Intervention Alters Rumen Microbial Community Development in Hu Lambs During Early Life. Front Microbiol.

[14] Jiao JZ, Huang JY, Zhou CS, Tan ZL (2015). Taxonomic Identification of Ruminal Epithelial Bacterial Diversity during Rumen Development in Goats. Appl Environ Microbiol.

[15] Seddik, H., Xu, L., Wang, Y. & Mao, S. Y. A rapid shift to high-grain diet results in dynamic changes in rumen epimural microbiome in sheep. *Animal*, 1-9 (2018). 10.1017/S1751731118003269 30560755

[16] Malmuthuge N, Liang GX, Guan LL (2019). Regulation of rumen development in neonatal ruminants through microbial metagenomes and host transcriptomes. Genome Biol.

[17] Petri RM, Kleefisch MT, Metzler-Zebeli BU, Zebeli Q, Klevenhusen F (2018). Changes in the rumen epithelial microbiota of cattle and host gene expression in response to alterations in dietary carbohydrate composition. Appl Environ Microbiol.

[18] Scharen M (2017). Alterations in the Rumen Liquid-, Particle- and Epithelium-Associated Microbiota of Dairy Cows during the Transition from a Silage- and Concentrate-Based Ration to Pasture in Spring. Front Microbiol.

[19] Jiao JZ (2019). Linkages between Epithelial Microbiota and Host Transcriptome in the Ileum during High-Grain Challenges: Implications for Gut Homeostasis in Goats. J Agr Food Chem.

[20] Jing XP (2018). Dietary supplements during the cold season increase rumen microbial abundance and improve rumen epithelium development in Tibetan sheep. J Anim Sci.

[21] Lv X (2019). The Signature Microbiota Drive Rumen Function Shifts in Goat Kids Introduced to Solid Diet Regimes. Microorganisms.

[22] McCann JC, Wickersham TA, Loor JJ (2014). High-throughput Methods Redefine the Rumen Microbiome and Its Relationship with Nutrition and Metabolism. Bioinform Biol Insights.

[23] Makkar HPS, Sharma OP, Dawra RK, Negi SS (1982). Simple Determination of Microbial Protein in Rumen Liquor. J Dairy Sci.

[24] Jiao JZ (2014). *In vitro* evaluation on neutral detergent fiber and cellulose digestion by post-ruminal microorganisms in goats. J Sci Food Agr.

[25] Kozich JJ, Westcott SL, Baxter NT, Highlander SK, Schloss PD (2013). Development of a dual-index sequencing strategy and curation pipeline for analyzing amplicon sequence data on the MiSeq Illumina sequencing platform. Appl Environ Microbiol.

[26] Pruesse E (2007). SILVA: a comprehensive online resource for quality checked and aligned ribosomal RNA sequence data compatible with ARB. Nucleic Acids Res.

[27] Cole JR (2009). The Ribosomal Database Project: improved alignments and new tools for rRNA analysis. Nucleic Acids Res.

[28] Kim D, Langmead B, Salzberg SL (2015). HISAT: a fast spliced aligner with low memory requirements. Nat Methods.

[29] Wang LK, Feng ZX, Wang X, Wang XW, Zhang XG (2010). DEGseq: an R package for identifying differentially expressed genes from RNA-seq data. Bioinformatics.

[30] Benjamini Y, Hochberg Y (1995). Controlling the False Discovery Rate - a Practical and Powerful Approach to Multiple Testing. J R Stat Soc B.

[31] Huang DW, Sherman BT, Lempicki RA (2009). Systematic and integrative analysis of large gene lists using DAVID bioinformatics resources. Nat Protoc.

[32] (2019). NCBI Sequence Read Archive.

[33] (2019). NCBI Sequence Read Archive.

[34] *ProteomeXchange Consortium* https://www.iprox.cn/page/project.html?id=IPX0007706000 (2023).

